# *Novakomyces olei* sp. nov., the First Member of a Novel Taphrinomycotina Lineage

**DOI:** 10.3390/microorganisms9020301

**Published:** 2021-02-02

**Authors:** Neža Čadež, Dénes Dlauchy, Miha Tome, Gábor Péter

**Affiliations:** 1 Food Science and Technology Department, Biotechnical Faculty, University of Ljubljana, Jamnikarjeva 101, 1000 Ljubljana, Slovenia; neza.cadez@bf.uni-lj.si (N.Č.); miha.tome@bf.uni-lj.si (M.T.); 2 National Collection of Agricultural and Industrial Microorganisms, Faculty of Food Science, Szent István University, Somlói út 14-16, H-1118 Budapest, Hungary; dlauchy.denes@szie.hu

**Keywords:** genome sequence, phylogenomics, functional gene analysis, novel yeast species, novel class, olive oil

## Abstract

Taphrinomycotina is the smallest subphylum of the phylum Ascomycota. It is an assemblage of distantly related early diverging lineages of the phylum, comprising organisms with divergent morphology and ecology; however, phylogenomic analyses support its monophyly. In this study, we report the isolation of a yeast strain, which could not be assigned to any of the currently recognised five classes of Taphrinomycotina. The strain of the novel budding species was recovered from extra virgin olive oil and characterised phenotypically by standard methods. The ultrastructure of the cell wall was investigated by transmission electron microscopy. Comparisons of barcoding DNA sequences indicated that the investigated strain is not closely related to any known organism. Tentative phylogenetic placement was achieved by maximum-likelihood analysis of the D1/D2 domain of the nuclear LSU rRNA gene. The genome of the investigated strain was sequenced, assembled, and annotated. Phylogenomic analyses placed it next to the fission *Schizosaccharomyces* species. To accommodate the novel species, *Novakomyces olei*, a novel genus *Novakomyces*, a novel family Novakomycetaceae, a novel order Novakomycetales, and a novel class Novakomycetes is proposed as well. Functional analysis of genes missing in *N. olei* in comparison to *Schizosaccharomyces pombe* revealed that they are biased towards biosynthesis of complex organic molecules, regulation of mRNA, and the electron transport chain. Correlating the genome content and physiology among species of Taphrinomycotina revealed some discordance between pheno- and genotype. *N. olei* produced ascospores in axenic culture preceded by conjugation between two cells. We confirmed that *N. olei* is a primary homothallic species lacking genes for different mating types.

## 1. Introduction

Taphrinomycotina, introduced by Eriksson & Winka [1], is one of the three subphyla of Ascomycota [2] and it is an assemblage of early diverging lineages of the phylum [3]. Taphrinomycotina is a sister group of the remaining Ascomycota (Saccharomycotina and Pezizomycotina) [4]. Before the introduction of Taphrinomycotina, the class Archiascomycetes had been proposed for the same group of fungi [5]. Currently, five classes are assigned to Taphrinomycotina, viz. Archaeorhizomycetes, Neolectomycetes, Pneumocystidomycetes, Schizosaccharomycetes, and Taphrinomycetes [6,7]. Each class includes a single order and except for Taphrinales each order is also monotypic. According to single locus or multigene phylogenetic analyses, the fungi assigned to Taphrinomycotina form either mono- or paraphyletic groups depending on the taxon sampling, the analysed phylogenetic markers, and the applied algorithm [3]. However, phylogenomic analyses support the monophyly of the taxa of Taphrinomycotina involved in the analyses (e.g., [8,9,10,11,12]).

The fungi assigned to Taphrinomycotina form a diverse group of organisms considering their morphology and ecology [3]. The asexual reproduction of *Schizosaccharomyces* (family Schizosaccharomycetaceae, order Schizosaccharomycetales, class Schizosaccharomycetes) and *Pneumocystis* (family Pneumocystidaceae, order Pneumocystidales, class Pneumocystidomycetes) species proceeds by fission. *Schizosaccharomyces* species are often found in sugar-rich substrates like fruits and honey, while *Pneumocystis* species are restricted to lung tissues and infect humans and some other mammals [3,13,14].

Taphrinomycotina species with a budding yeast-like state are currently assigned to the subordinate taxa of Taphrinomycetes. The most common genera are the dimorphic plant pathogenic *Taphrina* (family Taphrinaceae, order Taphrinales) and *Protomyces* (family Protomycetaceae, order Taphrinales). Their filamentous ascosporic state is restricted to the host plant tissues, while they reproduce by budding in culture [15,16]. The genus *Saitoella* (family Protomycetaceae, order Taphrinales) contains merely two described species, which are believed to be saprophytic. *Saitoella complicata*, the type species of the genus, was recovered from soil [17], and *S. coloradoensis* from insect frass [18]. Asexually, they reproduce by multilateral budding, but a sexual state is not known. Although, as a result of an 18S rRNA gene sequence analysis of fungi assigned to “Archiascomycetes”, Sjamsuridzal et al. [19] reported that “*S. complicata* groups with the apothecial ascomycete *Neolecta vitellina*”, *Saitoella* is currently assigned to family Protomycetaceae [20,21]. However, it was also noted that the evolutionary relationships of *Saitoella* within Taphrinomycotina remain uncertain [21]. In addition to *Protomyces* and *Saitoella*, some less investigated plant pathogenic genera; *Burenia*, *Protomycopsis*, *Taphridium*, and *Volkartia*, are also assigned to the family Protomycetaceae [22]. Currently, no nucleotide sequences corresponding to these genera are available from GenBank [23]; therefore, their placement among the members of family Protomycetaceae remains to be determined.

Unlike the above-noted fungi, *Neolecta* (family Neolectaceae, order Neolectales, class Neolectomycetes) accommodates four filamentous fruiting body-forming species, which form clavate stalked apothecia. The fruiting body formation by *Neolecta* species is unique among the known members of Taphrinomycotina and their placement among less complex fungi is an evolutionary enigma [3]. The genome of *Neolecta* is atypical among complex multicellular fungi. Contrary to the typical 10,000 genes encoded by them, *N. irregularis* is predicted to contain only a bit more than 5500 protein coding genes [24], thus realizing a complex multicellular life with smaller protein-coding capacity than *Saccharomyces cerevisiae* [25].

The most recently revealed lineage of Taphrinomycotina is the class Archaeorhizomycetes. The single genus *Archaeorhizomyces* (family Archaeorhizomycetaceae, order Archaeorhizomycetales) accommodates only two described species *A. finlayi* [26] and *A. borealis* [27]. *A. finlayi* was the first cultivated species of the Soil Clone Group I (SCGI) [28,29], which has been known earlier only from environmental DNA sequences. Both described *Archaeorhizomyces* species are slow-growing filamentous organisms associated with roots of conifers and rhizosphere soil [26,27].

Taphrinomycotina is the smallest subphylum of Ascomycota. While Pezizomycotina and Saccharomycotina include over 63,000 and 1000 known species, respectively, Taphrinomycotina contains only about 140 described species [4]. However, it is not known whether the small number of species currently assigned to Taphrinomycotina reflects small species diversity or is a result of ineffective isolation strategies [21]. Some recent results adumbrate that Taphrinomycotina may be a much larger group of fungi than earlier suspected. Based on environmental DNA studies, it is predicted that class Archaeorhizomycetes itself contains some 500 species [27].

Olive oil is the most important vegetable oil used for human nutrition in the Mediterranean region [30]. Its consumption confers a wide range of health benefits [30,31,32]. It has been revealed only recently that freshly prepared olive oil may harbour rich microbiota, including yeasts [33]. The more than 20 yeasts species reported from this previously unexplored habitat are members of subphylum Saccharomycotina [30] and 7 of them have been described just recently as novel species [34,35,36,37,38]. Yeasts can exert either a favourable or adverse effect on the sensory characteristics of olive oil [30].

During our investigations aiming at the exploration of the yeast biota of olive oil, we isolated from extra virgin olive oil a yeast strain, which proved to be the first representative of a novel Taphrinomycotina lineage. Although, despite our herein described efforts, only one strain has been recovered, its taxonomic novelty prompted us to propose a novel species, *Novakomyces olei*, for the above-noted strain. As according to phylogenomic analyses, the novel species cannot be assigned to any of the currently recognized five classes of Taphrinomycotina, we also propose a novel genus, a novel family, a novel order, and a novel class to accommodate the novel species.

## 2. Materials and Methods

### 2.1. Isolation and Characterization

The strain (NCAIM Y.02187^T^) involved in this study was isolated from extra virgin olive oil originating from Spain using the method described earlier [36]. Briefly, 12.5 mL of olive oil and the same volume of sterile distilled water were transferred to a screw-capped 50-mL centrifuge tube. After tightening the cap, the tube was vigorously shaken then centrifuged with 9400× *g* for 5 min. The supernatant olive oil was removed by Pasteur pipette and after vortexing, the water phase was filtrated through a 0.45-μm pore size cellulose nitrate membrane (Sartorius, Göttingen, Germany). The membrane was placed on the surface of Rose-Bengal chloramphenicol (RBC) agar (MERCK 1.00467, Kenilworth, NJ, USA) and incubated at 25 °C in darkness for 7 days. The colony developed under these conditions was isolated and purified by repeated streaking on glucose-peptone-yeast extract (GPY) agar [39]. In addition to the National Collection of Agricultural and Industrial Microorganisms (NCAIM), Budapest, Hungary, the strain was deposited in the Collection of Industrial Microorganisms (ZIM), Ljubljana, Slovenia and in Westerdijk Fungal Biodiversity Institute (CBS), Utrecht, The Netherlands.

Phenotypic characterization of the isolated strain was carried out by standard methods [39]. Carbon-source assimilation tests were carried out in liquid culture twice at different times and if the two results were not in agreement, more repeats were done. Urease activity was determined using urea R broth (URB). Ascosporulation was investigated on acetate agar, corn meal agar (CMA), ‘Spezieller Nährstoffarmer agar (SNA), potato–dextrose agar (PDA), 2% malt extract agar (MEA), GPYA, yeast extract-malt extract (YM) agar, V8 agar, diluted (1:9) V8 agar, yeast-carbon base (YCB, Sigma-Aldrich, St. Louis, MO, USA) agar, and yeast-carbon base agar supplemented with 0.01% ammonium sulphate (YCBAS) [39,40]. The cultures were incubated at 15 and 25 °C and examined weekly by a light microscope for 3 weeks then monthly for 150 days. For ultrastructural study by transmission electron microscopy (TEM), the strain was grown in 5% malt extract for 3 days on a rotary shaker (25 °C, 150 rpm). Cells were harvested by centrifugation and washed twice with sterile water. They were embedded in 2% agar-agar, postfixed in 0.5% osmium tetroxide in 0.1M phosphate buffer (PB) for 30 min. After dehydration in ascending ethanol (EtOH) series and contrasting with 1% uranyl acetate for 1 h in 70% EtOH, samples were incubated in propylene oxide and infiltrated with Durcupan resin (Sigma, St. Louis, MO, USA ) and flat-mounted between sheets of Aclar (EMS, Hatfield, PA, USA) within glass slides. After polymerization at 60 °C for 24 h, 70-nm sections were cut, mounted on single-slot formware-coated copper grids, contrasted with lead citrate (Ultrostain II, Leica, Wetzlar, Germany), and examined in a JEOL TEM-1011 (Tokyo, Japan) electron microscope at 80 kV; images were collected with a Megaview 12-bit 1024 × 1024 CCD camera. Ubiquinone was extracted from freeze-dried cell mass and its composition was determined at a commercial facility (Creative Proteomics, Shirley, NY, USA) by ultra-performance liquid chromatography–high-resolution mass spectrometry (UPLC-MS).

### 2.2. DNA Amplification, Sequencing, and Phylogenetic Analysis

The D1/D2 domain of the LSU rRNA gene, the nearly entire SSU rRNA gene, and the ITS regions of the investigated strain were amplified as described previously [41,42,43], and sequenced at a commercial sequencing facility (Biomi Ltd., Gödöllő, Hungary). Sequence similarity searches were performed against the GenBank sequence database using the BLAST 2.10.1 database search program [44]. For tentative phylogenetic placement of *N. olei*, the DNA sequence of the D1/D2 domain of NCAIM Y.02187^T^ along with the corresponding sequences of related and reference species retrieved from GenBank (Table 1) were aligned with MUSCLE [45] and phylogenetic trees were constructed from the aligned dataset applying maximum-likelihood analysis using the MEGA version X [46]. Positions with gaps were excluded from the analysis. Bootstrap support [47] for the tree was determined from 1000 replications.

### 2.3. Genome Sequencing, Assembly, and Annotation

The genome of the type strain of *N. olei* was sequenced by using a combination of long- and short-read sequencing technologies of PacBio and Illumina, respectively. The genomic DNA was isolated according to the protocol published by Schwartz and Sherlock [48] with a few adaptations for isolation of DNA from non-*Saccharomyces* yeast. The DNA was isolated from 50 mL of culture of *N. olei* NCAIM Y.02187^T^ grown in YPD broth (Condalab, Madrid, Spain) for 48 h at 28 °C, 220 rpm. After washing the cells in 0.9 M sorbitol solution, 20 µL of Lyticase (30 mg/mL, Sigma-Aldrich, St. Louis, MO, USA) and 5 µL of β-mercaptoethanol were added to the cells. The incubation time was at least 8 h at 37 °C. The formation of protoplasts was checked under the microscope. Following phenol/chloroform (Millipore, Burlington, MA, USA) extraction, the DNA was precipitated using 40 µL of 3 M sodium acetate (pH 5.5) and 1 mL of absolute ethanol and spooled with a pipette tip and resuspended in 200 µL of TE buffer.

Sequencing libraries were constructed using the 20-kb SMRTbell TPK (PacBio, Menlo Park, CA, USA) and TruSeq DNA PCR Free (350) (Illumina, San Diego, CA, USA) kits. The sequencing runs were performed on PacBio RSII and Illumina NovaSeq instruments at the Macrogen Europe B.V. (Amsterdam, The Netherlands) sequencing facility. The sequencing data are available at NCBI (PRJNA561902).

To generate whole-genome assembly, PacBio and Illumina reads were processed using modular computational tool LRSDAY 1.6.0 [49] adapted for non-*S. cerevisiae* yeasts. First, long reads generated by PacBio technology with 86× coverage were used to perform de novo genome assembly using Canu 1.8 assembler [50]. Illumina reads were first normalised to 200× coverage using BBNorm then clipped and trimmed to remove sequencing adaptors using Trimmomatic-0.38 [51]. The cleaned reads were subsequently mapped to the raw long-read-based genome assembly using processed BAM file for correcting base-level errors of the long-read-based assembly. The completeness of the genome was evaluated using BUSCO v3.0.2 software [52].

To determine ploidy, we used nQuire software [53] to align next-generation sequencing reads in the BAM file to an assembled genome to determine base frequency distributions between a frequency of 20 and 80. Because haploid genomes lack biallelic sites, their base frequency distributions have peaks at high and low base frequencies and are depleted at positions with base frequencies near 50, producing a plot with a “smiley-face” pattern.

For genome annotation, the Maker genome annotation pipeline v3.01.02 [54] as implemented in LRSDAY was used. For the homology evidence for genome annotation, the proteomes of all NCBI reference fungal taxa and transcripts of *Schizosaccharomyces pombe*, *Taphrina deformans*, *Neolecta irregularis*, and *Pneumocystis carinii* were used. tRNA genes were also annotated via the tRNAscan-SE v1.3.1 [55] of the Maker pipeline. For soft-masking of repeats, RepeatMasker with the fungal RepBase repeat library was used. Further, three ab initio gene predictions were used with Maker pipeline, SNAP [56], GeneMark-ES v4.57 [57] trained for *N. olei* genome, and AUGUSTUS v3.3.2 [58] with “*Schizosaccharomyces*” as an Augustus pre-existing species model. All resulting gene models together with homology evidence and masked repeats were used to perform the final set of annotations for the genome.

### 2.4. Phylogenomic Analyses

For the phylogenomic reconstruction of the Taphrinomycotina subphylum, one representative from each species available in GenBank was downloaded from NCBI webpage [59]. Six taxa were also included from Saccharomycotina and Pezizomycotina, and two outgroup taxa from Ustilaginomycotina and Agaricomycotina subphyla. For the species that lacked annotation available in the GenBank, the EST and protein homology evidence-based genome annotation was performed using Maker pipeline v3.01.02 [54]. Further, the protein sequences of the conserved single-copy orthologous were identified by BUSCO v3.0.2 using the “Ascomycota” reference dataset. A total of 110 conserved genes present in all taxa were extracted, aligned, and trimmed using MAFFT v7.310 [60] and trimAl v1.4 [61] as part of the BUSCO USECASE genomic utilities pipeline [62]. The alignments were concatenated in a single data matrix (49,596 amino acids sites) used as an input for maximum likelihood (ML) phylogenetic inference with RAxML v8.2.11 [63]. The best-fitting phylogenetic model was determined for each of the 110 gene trees using ModelFinder option of the IQ-TREE v. 1.6.1 [64] program. Branch support was evaluated with 100 bootstrap replicates and with internode certainty (IC) in order to evaluate the degree of conflicting bipartitions among the 110 individual gene trees [65,66], both in RAxML. For IC calculations, the ML gene-based trees were constructed in RAxML with the best-fit model of amino acid substitutions, which were separately estimated for each gene dataset in IQ-TREE. The gene-based partitions were used to calculate the internode certainty (IC) values on the concatenated gene tree in RAxML (option“-f i”).

Alternatively, for inferring coalescent-based phylogeny, individual gene trees constructed with the best-fitting model in RAxML were summarised by Astral v.5.7.4 [67] to produce the “species” tree. The topological robustness of each gene tree was evaluated by 100 bootstrap replicates. The phylogenetic trees were visualized using FigTree v1.4.4.

### 2.5. Identification of Missing Genes Their Gene Ontology (GO) Enrichment

To determine the gene presence/absence in *N. olei* in comparison to genes present in *S. pombe*, we first searched for putative homologues of 5120 open reading frames of *S. pombe* (PomBase Genome Database [68], downloaded October 2020) in NCBI’s Reference Sequence Database for Fungi [69] using blastp search of NCBI’s BLAST+ v2.8.1 application and e-cutoff value of 0.001 as specified by Steenwyk et al. [70]. One-hundred top searches were used for alignment using MAFFT v7.310. The aligned outputs were used to build a hidden Markov model (HMM) profile using HMMBUILD module of HMMER v3.2.1. The obtained HMM profile was then used to search for each gene in *N. olei* using HMMSEARCH. To determine in which functional categories these genes clustered, we conducted a functional grouping of genes based on gene ontology (GO) annotations with AMIGO2, version 44.264 [71] using the PANTHER™ GO Slim using the *S. pombe* subset.

### 2.6. Genome Searches for Genes Involved in Assimilation of Sugars

To determine presence/absence and their copy number of genes related to carbon source assimilation profiles in the genome of *N. olei* and its relatives, we used an NCBI’s BLAST+, v2.8.1 blastp, and tblastx tools using query protein sequences from the *S. pombe* ATCC 24843 against subject databases built from the *Novakomyces* proteomes and transcriptomes. The e-value threshold was set to 10^−5^ to assign the copy number of each gene as recommended by Riley et al. [9].

## 3. Results and Discussion

### 3.1. Isolation and Occurrence

Only a single colony developed from 12.5 mL of olive oil. It was picked up and purified by repeated streaking on GPY agar. The low cultivable cell concentration (one cfu in 12.5 mL olive oil) of the novel species suggests that it is allochthonous in olive oil. Indeed, it must be uncommon in this substrate because we have not isolated additional strains of this species, although more than 100 olive oil samples, including Spanish ones, were processed in our laboratories and more than 200 yeast strains were isolated from them, which were identified based on the DNA sequences of their LSU rRNA gene D1/D2 domain. The isolation of additional conspecific strains and revealing of further substrates harbouring this species is still pending.

### 3.2. Ribosomal Gene Sequence Comparisons and Phylogenetic Placement Based on LSU Sequences

The nucleotide sequences for the D1/D2 domain of the LSU rRNA gene (MG250349), the partial SSU rRNA gene (MW024001), and the ITS region (MW023954) of the nuclear ribosomal gene cluster of strain NCAIM Y.02187^T^ were determined, and deposited in GenBank with the accession numbers indicated in parentheses. The results of BLAST searches [44] with these sequences indicated that the novel species is not closely related to any fungus represented in the GenBank database. The closest hits shared less than 90% and 91% sequence similarities along the D1/D2 region and the SSU rRNA gene, respectively, while in case of the ITS region, no more than 50% query coverage was allowed by BLASTN search, which means that due to the profound divergence in the ITS1 and ITS2, effectively only the 5.8S rRNA gene similarities were compared. Even in the conserved 5.8S rRNA gene, the closest match among cultivated fungal strains differed by more than 4% substitutions from the corresponding DNA sequence of NCAIM Y.02187^T^. Due to the high degree of divergence among the sequences determined during this study and the sequences of the corresponding loci available from the GenBank, no genus-level identification of strain NCAIM Y.02187^T^ could be achieved by any of the three barcoding sequences determined. However, in case of the BLAST searches of the D1/D2 sequence against the GenBank database, some *Saitoella* (subphylum Taphrinomycotina) sequences appeared among the closest hits.

**Figure 1:**
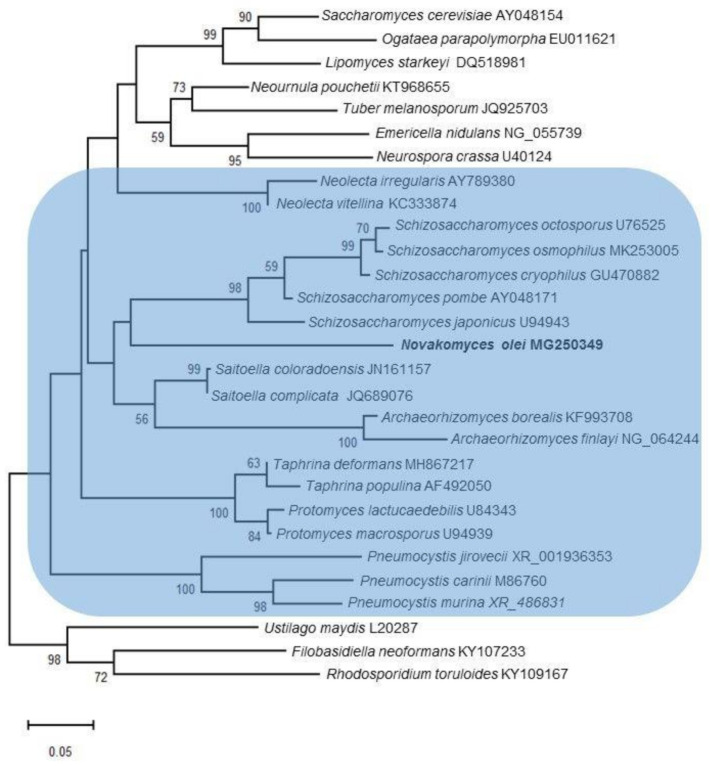
Phylogenetic placement of *Novakomyces olei* sp. nov. in the subphylum Taphrinomycotina using maximum-likelihood analysis and the Tamura–Nei model [73]. A discrete Gamma distribution was used to model evolutionary rate differences among sites. Analysis was performed based on the sequences of the LSU rRNA gene D1/D2 domain. Bootstrap percentages (1000 replicates) exceeding 50% are given at branch nodes. Bar, 5% nucleotide sequence divergence. *Ustilago maydis*, *Filobasidiella neoformans*, and *Rhodosporidium toruloides* were used as the designated outgroup species. Taphrinomycotina is highlighted by the blue background.

To achieve tentative placement of the novel species, D1/D2 trees were constructed with different taxon samplings using maximum-likelihood analysis. The placement of strain NCAIM Y.02187^T^ was inconsistent and dependent on the taxa considered, but it often formed a clade with *Schizosaccharomyces* species involved in the analyses. As an example, a phylogenetic tree is shown in Figure 1. The taxa were selected in such a manner so as to ensure that all the classes and families of Taphrinomycotina were represented. A few species from the subphyla Saccharomycotina and Pezizomycotina were also included in the analysis and one species per each subphylum of Basidiomycota were included as the (designated) outgroup. In the phylogenetic tree depicted in Figure 1, the strain NCAIM Y.02187^T^ is placed at the end of a long branch and occupies an early diverging position compared to the currently recognised *Schizosaccharomyces* species but without significant bootstrap support. Similarly, most of the basal lineages have no significant statistical support and the taxa of Taphrinomycotina, indicated by the blue background, form a paraphyletic group. This latter topology is consistent with some previous reports, i.e., the monophyly of the fungi assigned to Taphrinomycotina is not always supported by single and multilocus phylogenetic analyses [3,5,8,72].

Because of the inconsistent placement of strain NCAIM Y.02187^T^ and the absence of monophyly of the taxa assigned to Taphrinomycotina, we performed a robust phylogenomic analysis in order to determine the reliable placement of the novel species.

### 3.3. Phylogenomic Placement of the Novel Taxon

**Figure 2:**
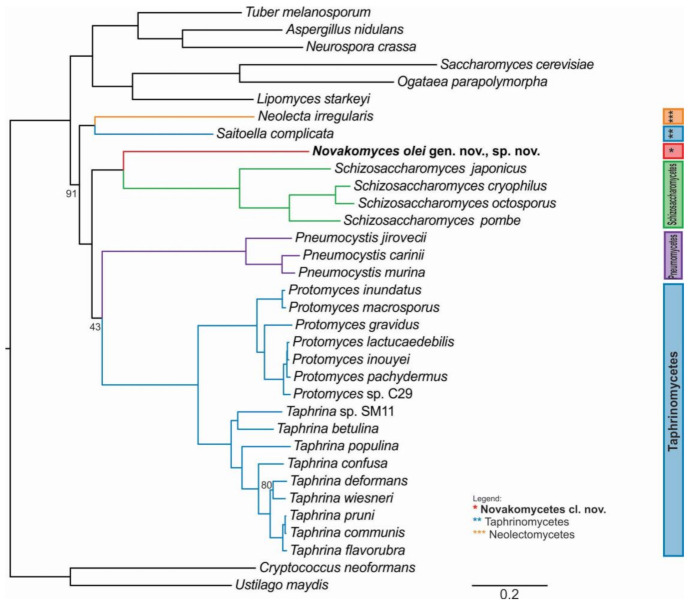
The phylogenetic relationships of Taphrinomycotina and related fungi based on concatenated alignment of 110 orthologous, single-copy BUSCO gene amino acid data matrix. The maximum likelihood (ML) phylogeny was reconstructed under the LG + GAMMA substitution model using RAxML as determined by IQ-TREE as the best model for a given dataset. Branch support values are indicated only for the nodes with <95% bootstrap support. Scale bar, number of amino acid substitutions per site. *Cryptococcus neoformans* and *Ustilago maydis* were the designated outgroup species in the analysis.

To infer a reliable phylogenetic position of the genetically divergent *N. olei* strain NCAIM Y.02187^T^, we sequenced, assembled, and annotated its genomic DNA. Furthermore, we used 26 publicly available genomes of taxa belonging to Taphrinomycotina and three genomes each representing subphyla of Saccharomycotina and Pezizomycotina and two outgroups from the fungal sister phylum Basidiomycota (Supplementary Materials Table S1). From 1315 BUSCO genes, we extracted 110 conserved single-copy orthologous genes common to all 34 taxa and performed a concatenation-based phylogeny on the basis of a single data matrix of 76,771 amino acid sites (Figure 2) and a coalescent-based phylogeny Supplementary Materials Figure S1). Both approaches yielded strongly supported and concordant phylogenies. Similar to the D1/D2 phylogenetic tree, *N. olei* NCAIM Y.02187^T^ is placed on a long branch basal to the *Schizosaccharomyces* species but with strong support (100% bootstrap, internode certainty (IC) of 0.95). In general, the phylogeny of Taphrinomycotina is divided into several well-supported clades comprising known classes. An exception is the class of Pneumocystidomycetes, which is a sister clade to the Taphrinomycetes in the concatenation-based phylogeny with only moderate support (43% bootstrap, IC of 0.00) but is placed in polytomy with Taphrinomycetes and with the clade comprising Schizosaccharomycetes and the new class Novakomycetes in the coalescence-based phylogenies, suggesting some conflicts between single-gene topologies of *Pneumocystis* species. This is likely due to their reduced genome and biased gene content and could be a consequence of their evolutionary adaptation to life exclusively in mammalian hosts [74].

The only taxon that is placed differently from its current taxonomic designation is the genus *Saitoella*, which is currently assigned to Protomycetaceae [21], but, in agreement with our phylogenomic analysis, its position as a sister group to *Neolecta* was found also by Sjamsuridzal et al. [19] but might be resolved differently with better taxon sampling.

### 3.4. Content of Novakozyma olei Genome

Using a combination of PacBio long-read and Illumina short-read sequencing technologies, we obtained a high-quality assembly of *Novakozyma olei* gen. nov., sp. nov. yielding a genome in a size of 14.3 Mb assembled into 15 scaffolds, which range from 0.17 to 2.6 Mb (Table 2). The G+C content of the new taxon is 43.8%, which is higher than the GC content of *S. pombe* (36%) and *P. carinii* (27.8%), confirming the evolutionary distance among these taxa (Supplementary Materials Table S2). For testing the ploidy status of *N. olei*, we used a base frequency plot (Supplementary Materials Figure S2), which peaked at high and low frequencies and as such lacks biallelic sites, suggesting a haploid genome. A similar situation was observed also in its sister clades as haploidy was found to be characteristic for 56 out of 57 strains of *S. pombe* [75] and in most trophic forms of the complex life cycle of *P. carinii* [76].

Even though the genome was assembled into long scaffolds with large overall size, only 80.9% (Table 2) of complete ascomycetous Benchmarking Universal Single-Copy Orthologues (BUSCO) genes were present. Similarly, after annotating the genome, only 3779 protein coding genes (Supplementary Materials Table S2) were found, which is only slightly higher than the number of genes (3646) found in obligate parasitic *P. carinii*, the type species of *Pneumocystis* [74].

### 3.5. Functional Analysis of Genes Missing in the Genome of *Novakomyces olei*

**Figure 3:**
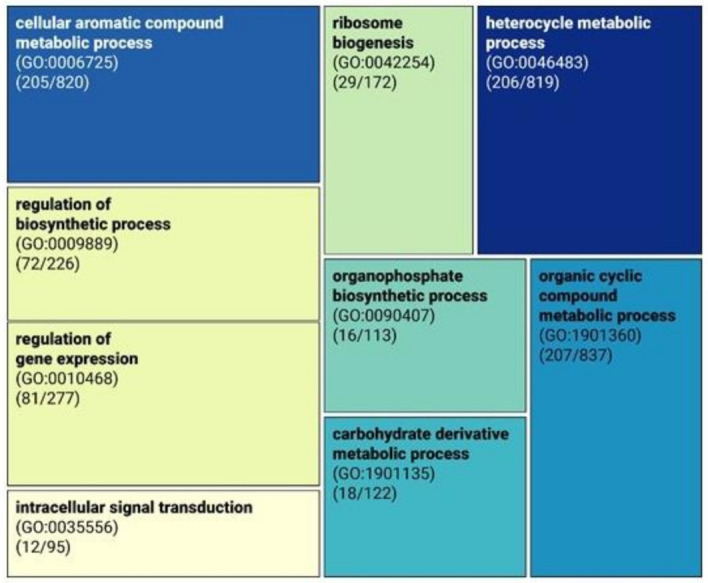
Significantly overrepresented slim gene ontology (GO) terms for biological processes of genes absent in *Novakomyces olei* sp. nov. compared to the reference gene set of *Schizosaccharomyces pombe* as its close relative. The sizes of the rectangles are proportional to the percentage of missing genes in a GO category.

Pervasive gene loss has mostly been correlated with a parasitic lifestyle, as is the case with *Pneumocystis* species, or with the ability to grow rapidly in a sugar-rich environment, as is the case with *Hanseniaspora* species [70,74]. Since gene loss was found mostly biased towards gene function [77,78], we assessed the presence/absence of genes in *N. olei* relative to *S. pombe*. We found that 1667 genes were missing in *N. olei*, and those were subjected to GO term enrichment to identify their putative functions. Significantly overrepresented GO-Slim terms for biological processes revealed categories related to the “cellular aromatic compound metabolic process” (GO:0006725, *p* < 0.001), “organic cyclic compound metabolic process” (GO:1901360, *p* < 0.001), and “heterocyclic metabolic process” (GO:0046483, *p* < 0.001), implying that *N. olei* may have reduced the metabolic capacity for the biosynthesis of complex organic molecules (Figure 3, Supplementary Materials Table S3). Therefore, we manually examined which of the missing genes are involved in the biosynthetic pathways for amino acids, vitamins, and nucleotides. In contrast to *Pneumocystis* species [74], most homologous genes for the biosynthesis of these important growth factors were present with few exceptions. The species lacks genes encoding enzymes involved in the metabolism of pyrimidine deoxyribonucleotides (*ccd1*, encoding cytidine deaminase) and biotin biosynthesis (*bio2*, encoding biotin synthase). The latter is consistent with its inability to grow in vitamin-free medium. However, the investigation of the primary metabolic pathways showed that they are not reduced to such an extent that the species would need to be dependent on any kind of symbiotic relationship as it is the case in *Pneumocystis*.

A large group of missing genes in *N. olei* (425 out of 1967 genes missing) were placed in a parent GO category “cellular processes” (GO:0009987, Supplementary Materials Table S3), many of which are involved in regulation processes, such as “regulation of biosynthetic process” (GO:0009889, 72 out of 226 genes missing) and “regulation of gene expression” (GO:0010468, 81 out of 277 genes missing) (Figure 3), which supports the prediction of Aravind et al. (2000) [79] that during the evolution, *S. pombe* acquired several genes that are also involved in the regulation of mRNA by lateral transfer. Based on this view, *N. olei* could serve as a missing link in studies on the evolution of fission and budding yeasts.

Additionally, examination of missing genes in GO-enriched categories “organophosphate biosynthetic process” (GO:0090407) and “intracellular signal transduction” (GO:0035556) revealed that *N. olei* lacks most of the genes involved in the biosynthetic pathway of cytochrome c oxidase (seven out of nine genes missing) and proton-transporting ATP synthase (five out of eight genes missing), which could imply that ATP production would be possible by substrate-level phosphorylation only, similarly as that observed for obligate intracellular parasite of microsporidia [80]. Nevertheless, further experimental evidence would be required for confirmation.

Lastly, we examined losses among the genes related to the sexual cell cycle. Under laboratory conditions, we were able to induce sporulation, which was sometimes preceded by conjugation between two cells of *N. olei*. By analysing the sequencing coverage across the whole genome, we were able to confirm the haploid state of the asexual cells of *N. olei* (Supplementary Materials Figure S2), which confirms that it is a primary homothallic species as the cells lack different mating types. From the genomic data of *N. olei*, we could confirm that the species lacks the gene *ste2* encoding a receptor for an alpha-factor pheromone, but at the same time, it harbours gene *ste3*, which encodes a receptor for a-factor pheromone. However, with our approach to determine genes based on their hidden Markov model (HMM) of protein profiles built from homologous genes from all reference fungal proteomes, we could not find any mating type genes that are homologous to *matPc*, *matPi*, *matMc*, and *matMi* of *S. pombe*. For this reason, we used the tBlastn search with a relaxed e-value threshold of 0.001, and the sequence query of the HMG box of *matMc* homologous to *P. carinii* [81]. We could find a nucleotide transcript of the HMG box in the genome of *N. olei* in the presence of *ste11*, a gene encoding DNA-binding transcription factor, *srm1* encoding spermidine synthase, and *rps20* encoding 40S ribosomal protein, however, without a conserved synteny with *S. pombe* or the two *Pneumocystis* species, *P. jirovecii* and *P. carini* [81].

### 3.6. Phenotypic Characters and Their Correlation with Genome Content

**Figure 4:**
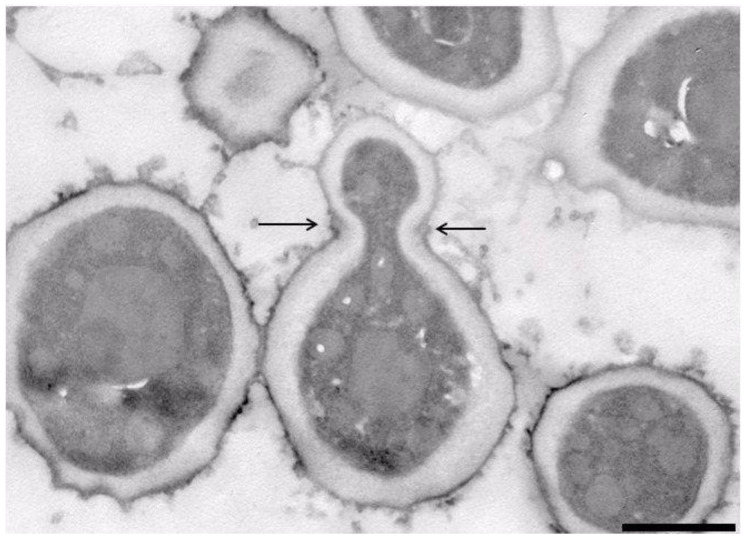
*Novakomyces olei* NCAIM Y.02187^T^. TEM micrograph of a section of a budding cell (5% malt extract, 3 days, 25 °C). Bud scar is indicated by arrows. Bar, 1000 nm. The image was taken by Bence Rácz.

**Figure 5:**
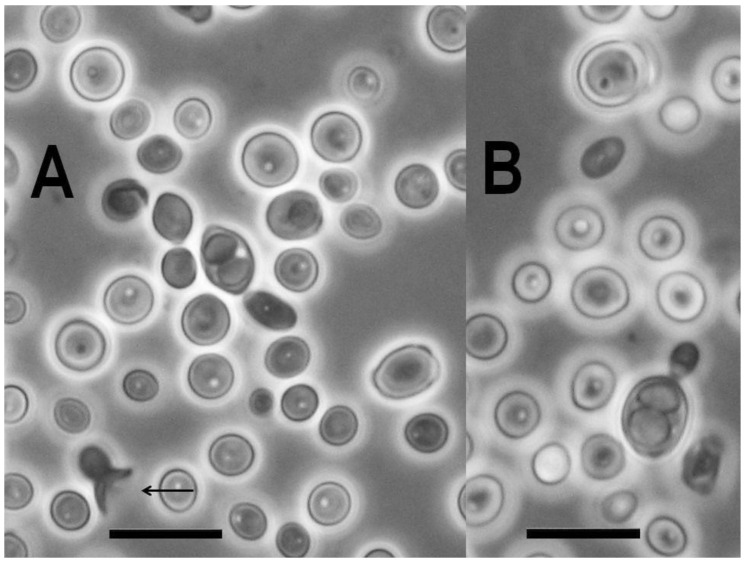
Ascosporulating culture of *Novakomyces olei* NCAIM Y.02187^T^. One two-spored ascus is shown in each panel. In panel (**A**), the remnant of an ascus formed by the conjugation of the mother cell and its bud is indicated by arrow. Bar, 10 μm for both panels (**A**,**B**).

The strain NCAIM Y.02187^T^ was characterised by standard methods [39]. The observed combination of urease activity and negative DBB colour reaction is characteristic for the fungi assigned to the subphylum Taphrinomycotina with budding yeast stage. Further, the two-layered cell wall coupled with enteroblastic budding (Figure 4) are characters shared with the genera *Taphrina, Protomyces,* and *Saitoella* [17,19,21]). The novel species differs from the above-noted three genera by the absence of the production of carotenoid pigments, from the genera *Taphrina* and *Saitoella* and most *Protomyces* species by its inability to assimilate nitrate and from *Saitoella* species by the formation of amyloid material (Table 3). Following a prolonged incubation period, exceeding 50 days, on PDA at 15 °C, strain NCAIM Y.02187^T^ formed 1–2 easily liberated subspheroid to ellipsoid ascospores sometimes preceded by heterogamous conjugation (Figure 5). The novel species is the first fungus having a budding yeast stage in Taphrinomycotina with documented ascosporulation in axenic culture. Like *Taphrina, Protomyces*, and *Saitoella* species, the novel species does not ferment sugars. Among the standard carbon-source assimilation tests, we observed several variable results, including the assimilation of glucose, which serves as a positive control for carbon-assimilation assay. Variable results for carbon-source assimilation tests have been documented in the case of several yeast species even if single strain per species was studied [82]; however, a variable result for glucose assimilation is unexpected.

**Figure 6:**
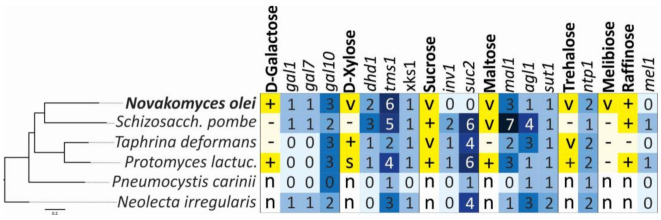
Correlation between assimilation profiles and gene content of *Novakomyces olei* and related species of Taphrinomycotina. Assimilation responses are represented by +, positive; −, negative; v, variable; s, slow; n, not determined (from Kurtzman et al. [82] and this study). Numbered boxes with blue colour intensity indicate the gene copy number determined by tBLASTn analyses. The maximum likelihood (ML) phylogeny was reconstructed under LG + GAMMA substitution model using RAxML. The scale bar presents amino acids substitutions per site.

In addition to the characterization of standard phenotypic traits of the novel species, we analysed how these metabolic traits are genetically encoded across Taphrinomycotina (Figure 6). For ascomycetous yeasts, it was shown that genome content and metabolic capabilities are generally congruent [78,84,85]. However, we could not confirm this for Taphrinomycotina species. For example, *N. olei* can assimilate galactose, and we found that all homologous genes from the *S. cerevisiae* metabolic pathway were present but were not physically linked. On the other hand, *S. pombe* also possesses all three *GAL* homologues, although it cannot use galactose as a sole carbon source. Matsuzawa et al. [86] showed that these genes in *S. pombe* are located near the chromosomal terminus and are most likely repressed by gene silencing. Similar examples are found for *T. deformans,* a species that is unable to grow on maltose even though it has several copies of genes involved in its catabolism (Figure 6). On the contrary, *N. olei* is able to assimilate sucrose, melibiose, and raffinose, but the gene homologues *inv1*, *suc2*, and *mel1* were not found. It is very likely that the metabolism of these sugars uses pathways of sugar utilisation that have not yet been identified.

### 3.7. Taxonomy

The taxonomic novelty of strain NCAIM Y.02187^T^ prompted us to propose a novel species, *Novakomyces olei*, to accommodate the strain although no additional isolates have been recovered yet and therefore the primary habitat and the ecology of the novel species remains to be explored. Further, a robust phylogenomic analysis placed the novel species, reproducing asexually by budding, in an early diverging position compared to the genus *Schizosaccharomyces* and confirmed that it is not closely related to any currently recognised fungus assigned to Taphrinomycotina. Therefore, in addition to the proposal of a novel species, a novel genus, a novel family, a novel order, and a novel class are also proposed to accommodate the subordinate taxa.

#### 3.7.1. Novakomycetes Dlauchy, Péter & Čadež cl. nov.

MycoBank no.: 838640.

Member of Taphrinomycotina O.E. Erikss. & Winka.

Asci are either unconjugated or formed following conjugation of mother cell and its bud. Asci contain one or two subspheroid or ellipsoid ascospores and are deliquescent (Figure 5). Yeast cells are subspheroid or ellipsoid and asexual reproduction proceeds by multilateral budding. The cell wall consists of two layers and budding is enteroblastic (Figure 4). Pseudohyphae and true hyphae are absent. Urease is produced, Diazonium Blue B reaction is negative. The major ubiquinone is Q-10. Starch-like compounds are produced. According to phylogenomic analysis, Novakomycetes is a sister taxon of the class Schizosaccharomycetes.

The nomenclature of the class is based on the generic name *Novakomyces* Dlauchy, Péter & Čadež gen. nov.

Type order: Novakomycetales Dlauchy, Péter & Čadež ord. nov.

#### 3.7.2. Novakomycetales Dlauchy, Péter & Čadež ord. nov.

MycoBank no.: 838642.

Member of Novakomycetes Dlauchy, Péter & Čadež cl. nov.

The diagnosis of the order Novakomycetales is based on the description of the class Novakomycetes cl. nov. The nomenclature of the order is based on the generic name *Novakomyces* Dlauchy, Péter & Čadež gen. nov.

Type family: Novakomycetaceae Dlauchy, Péter & Čadež fam. nov.

#### 3.7.3. Novakomycetaceae Dlauchy, Péter & Čadež fam. nov.

MycoBank no.: 838645

Member of Novakomycetales Dlauchy, Péter & Čadež ord. nov.

The diagnosis of the family Novakomycetaceae is based on the description of the order Novakomycetales ord. nov. The nomenclature of the family is based on the generic name *Novakomyces* Dlauchy, Péter & Čadež gen. nov.

Type genus: *Novakomyces* Dlauchy, Péter & Čadež.

#### 3.7.4. Novakomyces Dlauchy, Péter & Čadež gen. nov.

MycoBank no.: 838646.

Member of Novakomycetaceae Dlauchy, Péter & Čadež fam. nov.

*Novakomyces* (No’va.ko.my.ces. N.L. *Novakomyces* nom. masc. sing. n. combination of personal name Novák and Gr. masc. n. μύκης, -ητος a mushroom). The genus is named in honour of E.K. Novák in recognition of his major contribution to the taxonomy of yeasts.

The diagnosis of the genus *Novakomyces* is based on the description of the family Novakomycetaceae fam. nov. In addition, the single known species does not ferment sugars and does not assimilate nitrate.

Type species: *Novakomyces olei* Dlauchy, Péter & Čadež sp. nov.

#### 3.7.5. *Novakomyces olei* Dlauchy, Péter & Čadež sp. nov.

MycoBank no.: 838647.

*Novakomyces olei* (o.le’i. L. gen. neutr. sing. n. oleum,-i, olive oil, referring to its origin).

After 3 days of incubation at 25 °C in 5% malt extract, sediment is present, pellicle is not formed. Cells are subspheroid to ellipsoid, sometimes with one pointed end. They occur singly or in pairs and measure 2.0–5.0 × 2.5–6.0 μm. Asexual reproduction proceeds by multilateral budding, but the majority of the buds occupy polar position. On 5% malt extract agar after 3 days at 25 °C, the streak culture is butyrous, semi-glistening, smooth and flat, and white to cream coloured. The margin is entire. On slide culture with corn meal agar after 7 days at 25 °C, neither pseudohyphae nor septate hyphae are formed. Ascospore formation is preceded by parent cell–bud conjugation or cells are transformed to asci without conjugation and one or two easily liberating subspheroid or ellipsoid ascospores are formed in each ascus (Figure 5). Extremely scattered ascosporulation was observed in cultures grown on PDA following at least 50 days of incubation at 15 °C. The presence of heterogamous conjugation suggests that the species is homothallic. Fermentation is absent. The carbon compounds assimilated are D-glucose (variable), sucrose (variable), raffinose, melibiose (variable), D-galactose (positive or slow), α,α-trehalose (variable), maltose (variable), methyl-α-D-glucoside (positive or latent), salicin (slow and variable), arbutin (slow and variable), L-sorbose (variable), D-xylose (variable), ethanol (variable), glycerol (variable), meso-erythritol (variable), xylitol, D-mannitol (positive or slow), D-glucitol (variable), myo-inositol (variable), succinate (latent and variable), citrate (variable), D-gluconate (variable), 2-keto-D-gluconate (variable), glucono-δ-lactone (variable) and N-acetyl-D-glucosamine (slow and variable), while no growth occurs on inulin, lactose, melezitose, starch, cellobiose, L-rhamnose, L-arabinose, D-arabinose, D-ribose, methanol, ribitol, galactitol, L-arabinitol, DL-lactate, D-glucuronate, D-galacturonate, saccharate, D-glucosamine, propane 1,2 diol, butane 2,3 diol, and hexadecane. Ethylamine hydrochloride, L-lysine, and creatine (variable) are assimilated; potassium nitrate, sodium nitrite, cadaverine dihydrochloride, creatinine, glucosamine (as nitrogen source), and imidazole are not assimilated.

Amyloid material is formed. Growth in vitamin-free medium is absent. Growth occurs at 30 °C and is absent at 35 °C. Growth with 0.01% cycloheximide and with 10% NaCl is absent. Weak growth occurs on 50% *w/w* glucose yeast extract agar but is absent with 60% *w/w* glucose yeast extract agar and with 1% acetic acid. No acid is produced on chalk agar. Urea is hydrolysed but colour reaction with Diazonium Blue B is negative. The major ubiquinone is Q-10.

Holotype: NCAIM Y.02187 deposited in the National Collection of Agricultural and Industrial Microorganisms, Budapest, Hungary; isotypes: CBS 16559 deposited in the Westerdijk Fungal Biodiversity Institute, Utrecht, The Netherlands; ZIM 3702 deposited in Culture Collection of Industrial Microorganisms, Ljubljana, Slovenia. All are permanently preserved in a metabolically inactive state. The type culture was isolated from olive oil from Spain, in 2013.

The BioProject number for raw genome sequencing reads is PRJNA561902 (BioSample SAMN14856565), and the GenBank accession number for the assembled genome is JADEYG000000000.

## Figures and Tables

**Table 1:** List of strains included in the phylogenetic analysis, and the GenBank/EMBL/DDBJ accession numbers of their D1/D2 LSU rRNA gene sequences.

Species Name	Strain/Specimen *	GenBank Accession Numbers
*Archaeorhizomyces borealis*	NS99-600^T^	KF993708
*Archaeorhizomyces finlayi*	CBS 128710^T^	NG_064244
*Emericella nidulans*	ATCC 10074^T^	NG_055739
*Filobasidiella neoformans*	CBS 132^T^	KY107233
*Lipomyces starkeyi*	NRRL Y-11557^T^	DQ518981
*Neolecta irregularis*	ZW-Geo79-Clark	AY789380
*Neolecta vitellina*	DJM1533^V^	KC333874
*Neournula pouchetii*	MO-205345^V^	KT968655
*Neurospora crassa*	NRRL 13141	U40124
*Novakomyces olei*	NCAIM Y.02187^T^	MG250349
*Ogataea parapolymorpha*	NRRL YB-1982^T^	EU011621
*Pneumocystis carinii*	/	M86760
*Pneumocystis jirovecii*	RU7	XR_001936353
*Pneumocystis murina*	B123	XR_486831
*Protomyces lactucaedebilis*	NRRL YB-4353^A^	U84343
*Protomyces macrosporus*	NRRL Y-12879^A^	U94939
*Rhodosporidium toruloides*	CBS 6016^T^	KY109167
*Saccharomyces cerevisiae*	NRRL Y-12632^T^	AY048154
*Saitoella coloradoensis*	NRRL YB-2330^T^	JN161157
*Saitoella complicata*	NRRL Y-17804^T^	JQ689076
*Schizosaccharomyces cryophilus*	OY26^T^	GU470882
*Schizosaccharomyces japonicus*	NRRL Y-1361^T^	U94943
*Schizosaccharomyces octosporus*	NRRL Y-855^T^	U76525
*Schizosaccharomyces osmophilus*	SZ134-FG-A^T^	MK253005
*Schizosaccharomyces pombe*	NRRL Y-12796^T^	AY048171
*Taphrina deformans*	CBS 356.35^T^	MH867217
*Taphrina populina*	CBS 337.55^T^	AF492050
*Tuber melanosporum*	GB200^V^	JQ925703
*Ustilago maydis*	CBS 504.76^A^	L20287

* T, Type strain or ex-type strain; A, Authentic strain; V, voucher specimen; /, unknown; NRRL, ARS Culture Collection, National Center for Agricultural Utilization Research, Peoria, IL USA; CBS, Westerdijk Fungal Biodiversity Institute, Utrecht, The Netherlands; ATCC, American Type Culture Collection, Manassas, VA, USA; NCAIM, National Collection of Agricultural and Industrial Micro-organisms, Budapest, Hungary.

**Table 2:** Genome statistics of the *Novakomyces olei* sp. nov.

Attribute	Value	% of Total
**Genome size (Mb)**	14.3	
GC content (%)	43.8%	
Assembly statistics		
Number of scaffolds	15	
N50 (kb)	1688	
L50	4	
Number of unknown bases (N)	0	
**Annotation statistics**		
Number of protein-coding genes	3779	
Gene length (median, bp)	1694	22.2% of genome size
Exons per gene (mean)	5.7	−
Exon length (median, bp)	1047	12.5% of genome size
Intron length (median, bp)	261	5.7% of genome size
rDNA loci	11	−
tRNA genes	75	−
**Repeats (bp)**	741,619	5.2% of genome size
**Class I:** Retrotransposons ^1^		
LTR transposons	622	7.7% of repeats
Non-LTR transposons (LINE, SINE)	177	2.2% of repeats
**Class II:** DNA transposons	269	3.3% of repeats
Simple repeats	5913	73.8% of repeats
A and GA-rich regions	976	12.1% of repeats
Other	63	0.8% of repeats
**Complete BUSCO orthologs** ^2^	1063	80.9% out of 1315
Duplicated BUSCOs	2	0.2%
Fragmented BUSCOs	75	5.7%
Missing BUSCOs	177	13.4%

^1^ LTR, long terminal repeat; LINE, long interspersed nuclear element; SINE, short interspersed nuclear element. ^2^ Ascomycota dataset.

**Table 3:** Some salient characteristics of Taphrinomycotina genera reproducing asexually by budding.

Genus	Urease	DBB	Starch Formation	CoQ	Carotenoid Pigments	Nitrate Assimilation	Fermentation	Sexual Reproduction in Pure Culture
*Novakomyces*	+	−	+	10	−	−	−	+
*Taphrina*	+	−	+	10	+	+	−	−
*Protomyces*	v	−	v	10	+	+ (−)	−	−
*Saitoella*	+	−	−	10	+	+	−	−

Data are from Fonseca and Rodrigues [15] (*Taphrina*); Kurtzman [16] (*Protomyces*); Sugiyama and Hamamoto [83]; Kurtzman and Robnett [18] (*Saitoella*), and from the present study (*Novakomyces*). v, variable.

## Data Availability

Data supporting results can be found at https://doi.org/10.6084/m9.figshare.13676518.v1.

## Supplementary Materials

The following are available online at https://www.mdpi.com/2076-2607/9/2/301/s1. Figure S1: The genome-based phylogenetic placement of *Novakomyces olei* within subphylum Taphrinomycotina. The coalescent-based phylogeny was reconstructed from 110 single-copy BUSCO gene trees using Astral v5.7.4. [39]. Internal branch support values are indicated as posterior probability values (only values < 1) and internode certainty (only IC values ≤ 0.01). Figure S2: Evidence of haploid genome of *Novakomyces olei.* Base frequency plot of *Novakomyces olei* calculated by nQuire v1 [53] to directly infer ploidy levels from Illumina reads mapped to the assembled genome as a reference. Table S1: Summary of taxa included in phylogenomic analyses (related to Figure 2 and Figure S1). Information including taxonomic placement, strain designation, assembly number and BUSCO genome assembly completeness is provided. Table S2: Comparison of genomic and molecular characteristics of *Novakomyces olei* gen. nov., sp. nov. and related representative taxa of Taphrinomycotina. Table S3: List of significantly enriched GO-Slim terms for biological processes of the genes absent in *Novakomyces olei* in comparison to *Schizosaccharomyces pombe.*
