# Supplementary figures and images for: Novakomyces olei sp. nov., the First Member of a Novel Taphrinomycotina Lineage
Source: Microorganisms. 2021 Feb 2;9(2):301. doi: 10.3390/microorganisms9020301 (PMC7912804; doi:10.3390/microorganisms9020301)

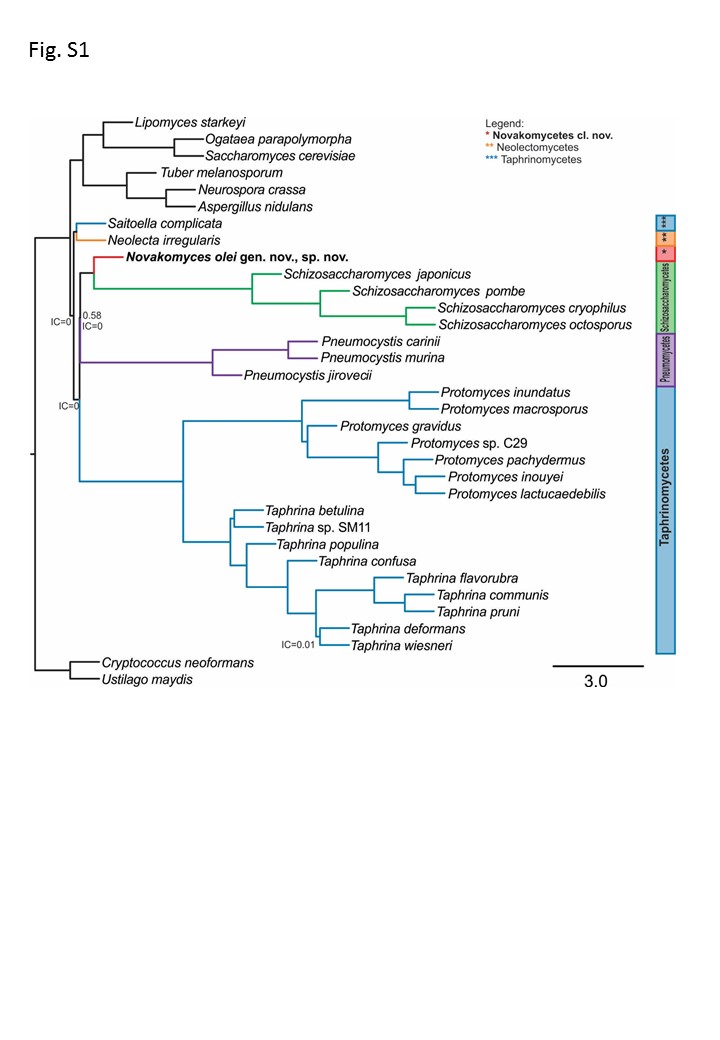

Supplement: Supplementary file 1 [file microorganisms-09-00301-s001.zip › Novakomyces_olei_Supplementary Figure 1.jpg]

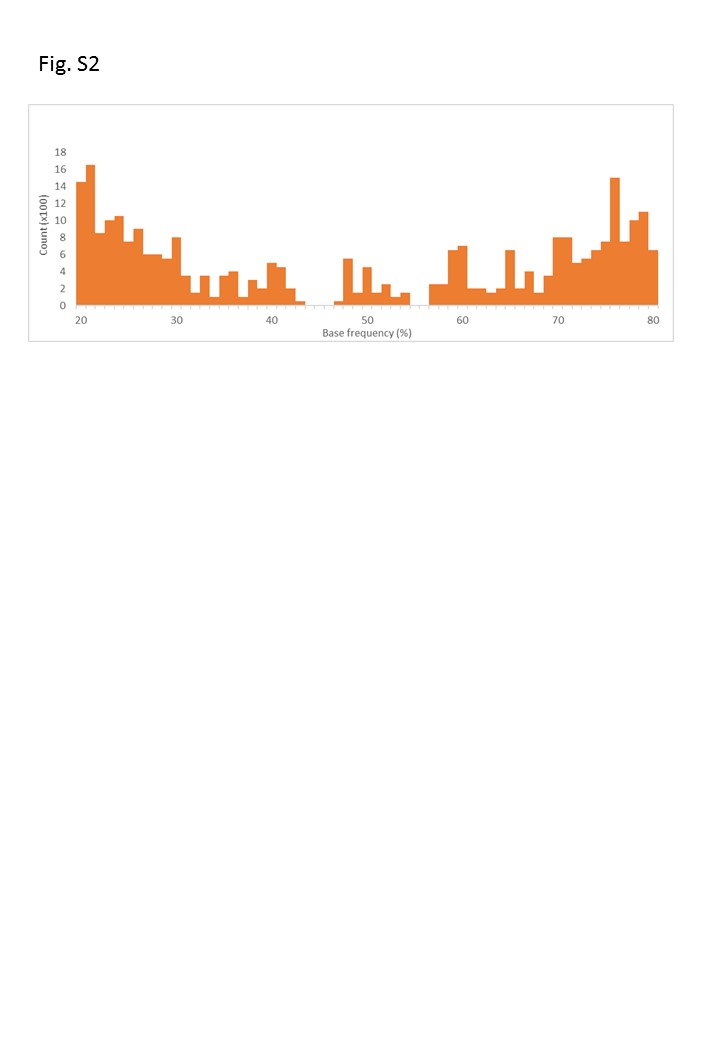

Supplement: Supplementary file 1 [file microorganisms-09-00301-s001.zip › Novakomyces_olei_Supplementary Figure 2.jpg]
